# SEVEN IN ABSENTIA Ubiquitin Ligases Positively Regulate Defense Against *Verticillium dahliae* in *Gossypium hirsutum*

**DOI:** 10.3389/fpls.2021.760520

**Published:** 2021-10-29

**Authors:** Zhongying Ren, Wei Liu, Xingxing Wang, Mingjiang Chen, Junjie Zhao, Fei Zhang, Hongjie Feng, Ji Liu, Daigang Yang, Xiongfeng Ma, Wei Li

**Affiliations:** ^1^State Key Laboratory of Cotton Biology, Key Laboratory of Biological and Genetic Breeding of Cotton of the Ministry of Agriculture and Rural Affairs, Institute of Cotton Research, Chinese Academy of Agricultural Sciences, Anyang, China; ^2^Collaborative Innovation Center of Henan Grain Crops, Agronomy College, Henan Agricultural University, Zhengzhou, China; ^3^Zhengzhou Research Base, State Key Laboratory of Cotton Biology, School of Agricultural Sciences, Zhengzhou University, Zhengzhou, China; ^4^State Key Laboratory of Plant Genomics, National Center for Plant Gene Research, Institute of Genetics and Developmental Biology, Innovation Academy for Seed Design, Chinese Academy of Sciences, Beijing, China

**Keywords:** cotton, ubiquitination, SINA, defense response, *Verticillium dahliae*

## Abstract

Ubiquitination is a post-translational regulatory mechanism that controls a variety of biological processes in plants. The E3 ligases confer specificity by recognizing target proteins for ubiquitination. Here, we identified SEVEN IN ABSENTIA (SINA) ubiquitin ligases, which belong to the RING-type E3 ligase family, in upland cotton (*Gossypium hirsutum*). Twenty-four *GhSINA*s were characterized, and the expression levels of *GhSINA7*, *GhSINA8*, and *GhSINA9* were upregulated at 24 h after inoculation with *Verticillium dahliae*. *In vitro* ubiquitination assays indicated that the three GhSINAs possessed E3 ubiquitin ligase activities. Transient expression in *Nicotiana benthamiana* leaves showed that they localized to the nucleus. And yeast two-hybrid (Y2H) screening revealed that they could interact with each other. The ectopic overexpression of *GhSINA7*, *GhSINA8*, and *GhSINA9* independently in *Arabidopsis thaliana* resulted in increased tolerance to *V. dahliae*, while individual knockdowns of *GhSINA7*, *GhSINA8*, and *GhSINA9* compromised cotton resistance to the pathogen. Thus, *GhSINA7*, *GhSINA8*, and *GhSINA9* act as positive regulators of defense responses against *V. dahliae* in cotton plants.

## Introduction

*Verticillium dahliae*, a soil-borne fungal pathogen, causes *Verticillium* wilt, which is a destructive vascular disease affecting more than 200 plant species, including agro-economically important cotton (BejaranoAlcazar et al., 1996; Fradin and Thomma, 2006; Cai et al., 2009). *V. dahliae* is notoriously difficult to control because of its strongly invasive pathogenicity and its ability to persist in soil, allowing it to penetrate host root xylem vessels and vascular tissues, which severely block plant vessels and eventually lead to plant stunting and wilt (Daayf et al., 1997; Klosterman et al., 2009). Additionally, the fungus has a broad host range and may survive in soil for several years, even in the absence of hosts (Klimes et al., 2015). In cotton production, the breeding of disease-resistant cultivars is an effective and practical management strategy to control the *V. dahliae* threat. However, owing to limited natural resources resistant to *V. dahliae*, using conventional approaches to breed resistant cultivars is challenging (Aguado et al., 2008; Jiang et al., 2009; Zhao et al., 2014; Klimes et al., 2015). In recent years, genetic engineering has become a promosing and environmentally friendly strategy to cope with *V. dahliae*, based on the characterization of cotton resistant candidate genes, like *GhMYB1*, *GbSOBIR1*, *GhCRR1*, *GbTSA1*, and *GhWAK7A* (Cheng et al., 2016; Han et al., 2019; Miao et al., 2019; Zhou et al., 2019; Wang et al., 2020).

The ubiquitin-proteasome system (UPS) plays significant roles in plant development and defense responses to both physiological and environmental stresses (Devoto et al., 2003; Mukhopadhyay and Riezman, 2007; Tommer and Mark, 2008; Santner and Estelle, 2010). The ubiquitination process usually occurs through the sequential actions of three enzyme types, ubiquitin-activating enzymes (E1s), ubiquitin-conjugating enzymes (E2s) and ubiquitin ligases (E3s), which co-ordinately attach ubiquitin to candidate protein substrates for targeted degradation through the 26S proteasome degradation pathway (Pickart, 2001; Kraft et al., 2005; Harper and Schulman, 2006). The ubiquitin E1s and E2s are relatively conserved, but the E3s are very diverse, because they are responsible for recruiting specific target proteins for ubiquitination. On the basis of subunit composition, the E3 ubiquitin ligases may be classified into single- and multi-subunit groups. The HECT and RING/U-box E3 ligases function as single subunits, whereas the SCF and anaphase-promoting complex E3 ubiquitin ligases consist of multiple polypeptides (Vierstra, 2003; Chen and Hellmann, 2013). Through their subunit compositions, E3 ligases determine the specificity of the candidate substrates for ubiquitination (Vierstra, 2009; Shimizu et al., 2010; Pepper et al., 2017).

The SEVEN IN ABSENTIA (SINA) proteins are RING-type E3 ligases that contain an N-terminal-located RING finger domain, followed by the conserved SINA domain that is involved in substrate recognition and dimerization (Welsch et al., 2007; Den Herder et al., 2008). The originally identified SINA E3 ligase in *Drosophila melanogaster* regulates photoreceptor differentiation (Carthew and Rubin, 1990). SINA homologs play critical roles in animals, including tumor suppression, apoptosis, leukemogenesis, hypoxia responses and autoimmunity (Christian et al., 2011; Kramer et al., 2013; Ma et al., 2015; Rajsbaum et al., 2015; Feng et al., 2019). Likewise, SINA E3 ligases are involved in various plant developmental stages and several stress responses (Mazzucotelli et al., 2006; Santner and Estelle, 2010). In *Arabidopsis thaliana*, SINAT2 interact with AtRAP2.2 to mediate the carotenogenesis of leaves (Welsch et al., 2007). SINAT1 and SINAT2 interact with the autophagy-related protein ATG6 to regulate the autophagy pathway (Qi et al., 2017). In rice (*Oryza sativa*), RNA interference silencing of drought-induced *SINA* gene 1 (*OsDIS1*) enhances drought tolerance (Ning et al., 2011). In tomato (*Solanum lycopersicum*), the overexpression of *SlSINA4* activates the defense-related cell death signaling (Wang et al., 2018). In banana (*Musa acuminate*) fruit, MaSINA1 negatively participates in cold-stress responses by mediating the stability of MaICE1 (Fan et al., 2017). In apple (*Malus* × *domestica*) calli, MdSINA2 increases the sensitivity to ABA stress (Li et al., 2020). In wheat (*Triticum aestivum*), the TaSINA E3 ligase increases the biomass and yield under heat-stress conditions (Thomelin et al., 2021). However, the functions and applications of SINA E3 ligases associated with *Verticillium* wilt resistance remain unknown.

To investigate the functions of E3 ubiquitin ligase genes in response to *V. dahliae* in upland cotton (*Gossypium hirsutum*), we identified three SINA E3 ligase genes, *GhSINA7*, *GhSINA8*, and *GhSINA9*, which are induced by *V. dahliae* infections. The overexpression of each of these genes conferred enhanced tolerance to *V. dahliae* in the transgenic Arabidopsis, whereas the silencing of each gene inhibited the defense capabilities against pathogen infection. These findings indicate the participation of SINA E3 ligases in plant defense against fungal pathogens and provide effective gene resources for the development of *Verticillium* wilt-resistant cotton cultivars.

## Materials and Methods

### Plant Materials and Growth Conditions

The seeds of *G. hirsutum* cv. Zhongzhimian No. 2 (resistant cultivar) were sown in sterile mixed soil (vermiculite:humus = 1:1) in a greenhouse at 28°C under 16-h light/8-h dark conditions.

The seeds of *A. thaliana* ecotype Col-0 were sown on Murashige and Skoog (MS) medium, and then, the seedlings were planted in pots containing mixed soil (vermiculite:humus = 1:1) in a culturing room at 23°C under 16-h light/8-h dark conditions.

The *Nicotiana benthamiana* seedlings were grown in pots containing mixed soil (vermiculite:humus = 1:1) at 25°C for approximately 6 weeks, under 16-h light/8-h dark conditions in a greenhouse.

### Pathogen Preparation and Inoculation Treatments

The defoliating isolate V991 of *V. dahliae* was grown on potato dextrose agar for 4 days at 25 °C and then further cultured in liquid Czapek medium for another 5 days. The spores were collected and resuspended in deionized water. For *V. dahliae* infections, the roots of cotton seedlings grown under hydroponic conditions for 2 weeks were inoculated with a spore suspension (10^6^ spores ml^–1^), and harvested after 24 h for RNA extraction. For the inoculation of *SINA*-silenced cotton plants, the spore concentration was adjusted to the 10^6^ spores ml^–1^ and injected into the hypocotyl at 1 cm below the cotyledons using a springe needle, approximately 3 μl per plant (Bolek et al., 2005). For Arabidopsis infections, 18-old seedlings were gently uprooted from soil, rinsed in sterile water and the roots were dip-infected with a *V. dahliae* spore suspension (4 × 10^5^ conidia ml^–1^) for 2 min. Then, the plants were transferred into fresh steam-sterilized soil for the detection of disease symptoms.

### Disease Index Calculation, *V. dahliae* Recovery Assays and Splitting Stem Observations

The disease index (DI) was calculated using the following formula: DI = [(Σ disease grades × number of infected plants)/(total number of plants × 4)] × 100. After *V. dahliae* infection, seedlings were classified into five grades, 0, 1, 2, 3, and 4, on the basis of the disease severity as reported previously (Xu et al., 2014). Fungal recovery assays were performed as previously described (Fradin et al., 2009). Briefly, after surface sterilization, the first internode sections were cut into 3–5 mm slices from control and *SINA*-silenced cotton plants, and then, they were separately cultured on PDA medium. To determine the level of *V. dahliae* colonization, the longitudinal cross-sections of cotyledonary nodes were dissected and observed under a stereoscopic microscope (Leica, Wetzlar, Germany) (Fradin et al., 2009).

### Measurement of Fungal Biomass

For fungal biomass quantification, stems of inoculated cotton plants and roots of inoculated Arabidopsis plants were collected for DNA extraction. The internal transcribed spacer (ITS) region of ribosomal DNA was targeted using the fungus-specific ITS1-F primer in combination with the *V. dahliae*-specific reverse primer STVE1-R (Hu et al., 2018). Primers for cotton *Histone3* and Arabidopsis *Actin2* genes were used as endogenous plant controls. The quantitative real-time PCR (qRT-PCR) analysis was conducted on genomic DNA (Santhanam et al., 2013). The primer sequences are listed in Supplementary Table 1.

### Virus-Induced Gene Silencing in Cotton

*pTRV1* and *pTRV2* (Liu et al., 2002) vectors were used for Virus-Induced Gene Silencing (VIGS) experiments. The specific fragments of *GhSINA*s were amplified and inserted independently into *pTRV2*. The primer sequences are listed in Supplementary Table 1. The recombinant plasmids were transformed into *Agrobacterium tumefaciens* strain GV3101. *Agrobacterium* cultures (OD_600_ = 1) harboring the *pTRV1* and *pTRV2-GhSINA* plasmids were mixed at 1:1 ratios and infiltrated into two full cotyledons of 7-day-old cotton seedlings using a needleless syringe as described previously (Gao et al., 2011). Alternatively, cotyledons of seedlings were infected with the mixture by vacuum infiltration (Qu et al., 2012). The *G. hirsutum CLA1* gene was used as the positive control for the silencing system. Approximately 7 days after the *Agrobacterium*-mediated transformation of cotton plants, the *GhCLA1* gene-silenced plants displayed the photobleaching phenotype, suggesting that the VIGS experiment was performed well.

### *In vitro* Ubiquitination Assay

The full-length open reading frames (ORFs) of *GhSINA7*, *GhSINA8*, and *GhSINA9* genes were cloned independently into the pMAL-C2x vector to generate maltose binding protein (MBP)-fusion proteins. The primers used in the assay are listed in Supplementary Table 1. Recombinant proteins were expressed in the *Escherichia coli* BL21 strain, in the presence of 0.5 μM isopropyl β-D-1-thiogalactopyranoside, purified by affinity chromatography using amylose resin (NEB, Ipswich, MA, United States) and used for *in vitro* ubiquitination analyses as described previously (Xie et al., 2002; Cho et al., 2008; Zhao et al., 2013). Purified fusion MBP-SINAs (3 μg) were incubated independently in 30 μl ubiquitination reaction buffer (50 mM Tris-HCl, pH 7.5, 5 mM MgCl_2_, 2 mM DTT, and 2 mM ATP), 5 μg biotinylated-ubiquitin (Enzo, #BML-UW9920-0001), 100 ng E1 (Enzo, #BML-UW9920-0001) and 40 ng Human E2 (UbcH5b) at 30°C for 3 h (Enzo, #BML-UW9920-0001). The reactions were terminated by adding the 5 × sample buffer, and half of the mixtures were separated using 7.5% SDS-PAGE gel. The proteins were identified by western blotting using an anti-biotin antibody (Cell Signaling, 1:3,000 dilution). Images were visualized on Tanon-5200 Chemiluminescent Imaging System (AI600 UV, United States) using chemiluminescence following the manufacturer’s instructions (ECL; Amersham, Thermo).

### Yeast Two-Hybrid Assay

The Y2H screening was constructed in accordance with the instructions of the Matchmaker Gold Yeast Two-Hybrid (Y2H) System (Clontech, Palo Alto, CA, United States) (Bai and Elledge, 1996). The full-length cDNAs of the *GhSINA*s were cloned independently into both the bait vector pGBDK7 and the prey vector pGADT7. The constructs were co-transformed into yeast strain AH109, and the co-transformed yeast colonies were streaked onto SD/-Leu/-Trp DO (DDO) medium. After growth at 30°C for 72 h, independent colonies of the same size were transferred to SD/-Leu/-Trp/-Ade/-His DO (QDO) medium supplemented with X-α-Gal (Clontech) to assess the pair-wise interactions among the GhSINA proteins. The primers for the Y2H constructs are listed in Supplementary Table 1.

### RT-PCR and Quantitative Real-Time PCR Analyses

Total RNAs were extracted from different tissues of cotton or Arabidopsis plants using TRIzol reagent (TIANGEN, Beijing, China) and treated with DNase I in accordance with the manufacturer’s protocol. In total, 1 μg of total RNA was reverse transcribed using a cDNA synthesis kit, version R323 (Vazyme, Nanjing, China). The RT-PCR analyses were performed as described previously (Hu et al., 2018). The qRT-PCR assays were performed using SYBR Green Real-time PCR Master Mix (Vazyme) on a LightCycler480 system (Roche, Germany). PCR amplification parameters were as follows: 95°C for 30 s, followed by 40 cycles of 95°C for 5 s and 60°C for 30 s. The cotton *Histone3* or Arabidopsis *Actin2* gene was used as an internal control. The primers used in the RT-PCR and qRT-PCR are listed in Supplementary Table 1.

### Plasmid Construction and Arabidopsis Transformation

The ORFs of the *GhSINA*s were cloned and inserted independently into the vector pCambia2300 (CAMBIA) containing the *CAULIFLOWER MOSAIC VIRUS* (CaMV) *35S* constitutive promoter. The primer sequences are listed in Supplementary Table 1. All the constructs were confirmed by sequencing, and then introduced independently into *A. tumefaciens* GV3101. The Arabidopsis plants (ecotype Col-0) were transformed using the *A. tumefaciens*-mediated floral dip method (Zhang et al., 2006). Transgenic Arabidopsis were selected on MS medium containing kanamycin, and the selected transgenic seedlings were further screened using genomic PCR. Homozygous T_3_ transgenic lines were generated for the functional analysis.

### Subcellular Localization in *N. benthamiana*

The ORFs of *GhSINA7*, *GhSINA8*, and *GhSINA9* were fused independently to eGFP in the modified pCambia2300-eGFP expression vector. The primer sequences are listed in Supplementary Table 1. *Agrobacterium* cells (strain GV3101) containing recombinant plasmids were infiltrated into *N. benthamiana* leaves. The infiltrated plants were incubated for 48 h at 25°C under dark conditions. Fluorescence signals were then visualized with a confocal laser scanning microscope after three infiltrations (Olympus, Germany).

### Bioinformatics Analysis

The available genome data for three cotton species, *G. hirsutum* (AD1; ZJU assembly), *G. arboreum* (A2; CRI assembly) and *G. raimondii* (D5; JGI assembly), were collected from the CottonFGD database^1^. The amino acid sequences of five *A. thaliana* SINAs (AtSINAT1, AtSINAT2, AtSINAT3, AtSINAT4, and AtSINAT5) were used as queries to identify candidates in three cotton protein databases using the BlastP program. The *e*-value was set at 1e-10. Redundant sequences were removed. The candidates were then filtered to confirm the presence of the conserved RING finger (IPR013083) and SINA domains (PF03145.16; IPR004162) using the Pfam database^2^ and InterPro database^3^. Subsequently, the coding sequences of all putative SINA genes in *G. hirsutum* were further verified by cloning and sequencing. For the phylogenetic analysis, all the SINA protein sequences of cotton and Arabidopsis (Supplementary Table 2) were employed to construct an unrooted phylogenetic tree by the neighbor-joining method with 1,000 bootstrap replicates using the MEGA 7.0 software (Hall, 2013; Kumar et al., 2016). For the sequence logo analysis, a multiple sequence alignment was performed using the ClustalX 2.0 software (Larkin et al., 2007). The conserved RING finger and SINA domain sequences of upland cotton SINAs were aligned, and the results were used as the input file in the online tool WEBLOGO^4^ (Crooks et al., 2004).

## Results

### Identification of the SEVEN IN ABSENTIA Genes in *Gossypium* Species

**FIGURE 1:**
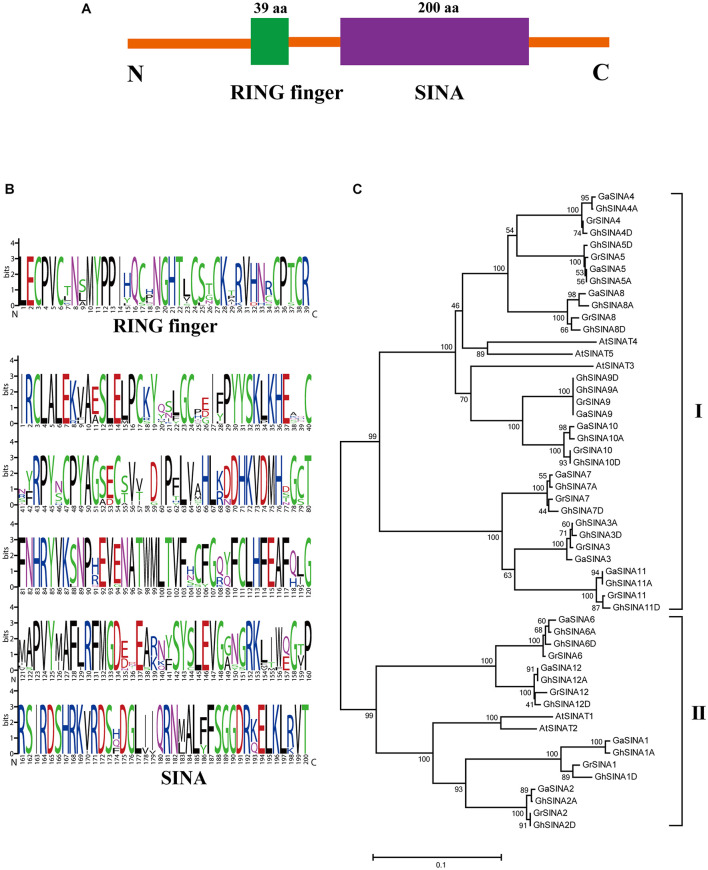
Conserved domain and phylogenetic analyses of SINA ubiquitin ligases in cotton. **(A)** Members of the *SINA* gene family are characterized by two highly conserved domains: a RING finger domain toward the N-terminal and a SINA domain toward the C-terminal. **(B)** Sequence logos showing the highly conserved RING finger and SINA domains in *G. hirsutum* SINA ubiquitin ligases. **(C)** Phylogenetic analysis of SINA ubiquitin ligases in *G. arboreum*, *G. raimondii*, *G. hirsutum* and Arabidopsis. The unrooted phylogenetic tree was generated with MEGA 7.0 using the neighbor-joining method with 1,000 bootstrap replicates. Numbers on the tree branches represent bootstrap values.

To investigate the roles of SINA ubiquitin ligases in cotton, 12 *G. arboreum*, 12 *G*. *raimondii* and 24 *G. hirsutum SINA* genes were identified in the cotton genome database. Then, all the *G. hirsutum SINA* genes were cloned and sequenced. As a result, the coding sequence of *GhSINA8* acquired from sequencing ten clones was shorter compared with its initial annotation in the genome database, while was similar with *GaSINA8* and *GrSINA8*. Therefore, the coding sequence of *GhSINA8* acquired from cloning was used in the study. These *SINA*s encoded two typically conserved domains: a RING finger domain (located toward the N-terminus) and a SINA domain (located toward the C-terminus) (Figure 1A). To examine the features of the homologous domain sequences and the conservation frequency of each residue within the RING finger and large SINA domains, multiple sequence alignments were conducted to generate sequence logos in cotton (Figure 1B). In general, the RING finger domain contained 39 conserved basic residues, whereas the basic region of the SINA domain had 200 conserved residues, which were responsible for interactions with specific target substrates. To further study the evolutionary relationships among SINA proteins in cotton (*G. hirsutum*, *G. arboreum*, and *G. raimondii*) and Arabidopsis, a phylogenetic tree was constructed. All the identified SINA proteins were divided into two groups, with 32 cotton *SINA* genes (8, 8, and 16 from *G*. *raimondii*, *G. arboreum*, and *G. hirsutum*, respectively) and 3 Arabidopsis *SINA* genes in Group I, and 16 cotton *SINA* genes (4, 4, and 8 from *G*. *raimondii*, *G. arboreum*, and *G. hirsutum*, respectively) and 2 Arabidopsis *SINA* genes in Group II (Figure 1C). Twice the number of SINA genes was found in upland cotton than in the diploid cottons *G. arboreum* (AA) and *G. raimondii* (DD), which is consistent with the allotetraploid cotton *G. hirsutum* (AADD)-producing polyploidization event being derived from the natural hybridization of diploid progenitors resembling *G. arboreum* and *G. raimondii*. The number of *SINA* genes is significantly higher in cotton than in Arabidopsis, indicating that *SINA* gene family members expanded distinctly in the ancestors of cotton and during cotton genome evolution.

### Expression Analysis of the *GhSINA* Genes in Response to *V. dahliae*

**FIGURE 2:**
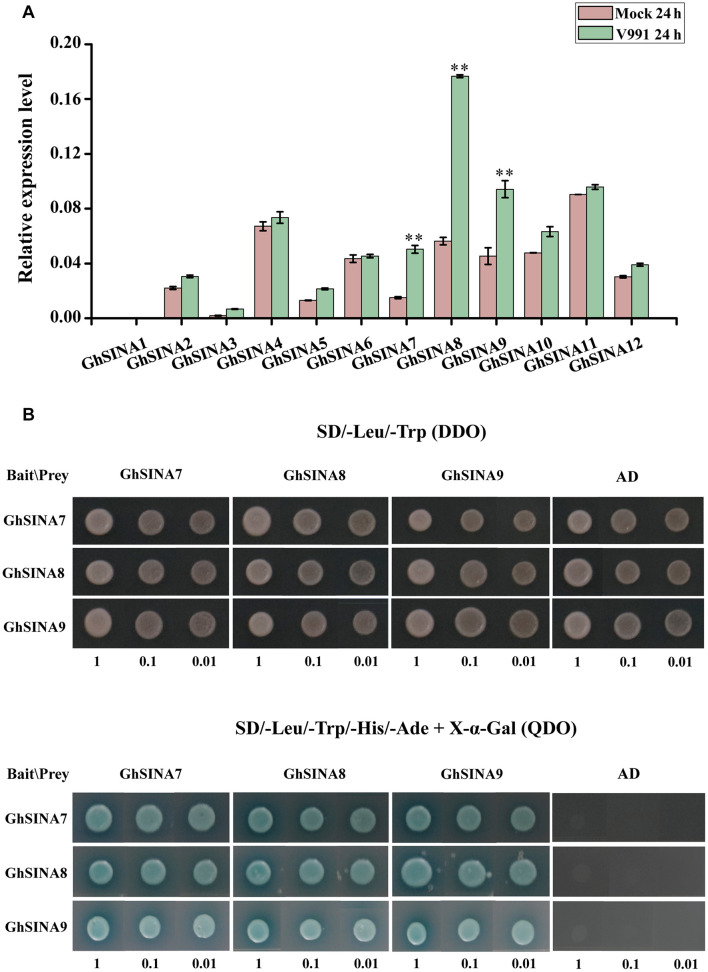
Expression and yeast two-hybrid analyses of *GhSINAs*. **(A)** Expression levels of *SINA* genes in the resistant cultivar *G. hirsutum* cv. Zhongzhimian No. 2 roots at 24 h after inoculation with *V. dahliae*. Total RNAs were extracted from roots of 14-day-old seedlings at 24 h after *V. dahliae* infection. Error bars represent the SDs of three biological replicates. Cotton *Histone3* was used as an internal control. Asterisks indicate statistically significant differences, as determined by Student’s *t*-test (^∗∗^*P* < 0.01). **(B)** Yeast two-hybrid assays detected pair-wise interactions among the three GhSINA proteins (GhSINA7, GhSINA8, and GhSINA9). Transformed yeast cells with 10-fold serial dilutions were grown on SD/-Leu/-Trp DO (DDO) mediums and SD/-Leu/-Trp/-Ade/-His DO (QDO) mediums (containing X-α-gal) media. GhSINAs-BD/AD were used as negative controls.

The germ tube germination and hyphal growth could be detected on the roots of cotton seedlings at 24 h after inoculation with GFP-labeled *V. dahliae* (Zhao et al., 2014; Li et al., 2016). To determine whether the expression levels of *GhSINA* genes changed after 24 h in response to *V. dahliae* infection, their transcript levels in the roots of the resistant upland cotton cultivar Zhongzhimian No. 2 were investigated. Because of the highly similar sequences of homoeologous gene pairs (similar values > 97%) (Supplementary Figure 1), it was difficult to differentiate between the homoeologs using quantitative real-time PCR (qRT-PCR). Consequently, they were amplified together. Overall, the accumulation level of the combined *GhSINA* gene transcripts was greater than the control (Figure 2A). Compared with the mock-inoculated controls, the expression levels of *GhSINA7*, *GhSINA8*, and *GhSINA9* were induced by approximately 3. 5-, 3. 2-, and 2.0-fold, respectively, at 24 h after pathogen inoculation. We then focused on of the roles of *GhSINA7*, *GhSINA8*, and *GhSINA9* in cotton defense against *V. dahliae*.

### Interactions Among GhSINA7, GhSINA8, and GhSINA9 Proteins

SINA proteins form homodimers as well as heterodimers to accomplish their biological functions (Den Herder et al., 2008; Yang et al., 2015). To determine whether the three candidate GhSINA proteins had the ability to undergo homo- and/or heterodimerization, the pair-wise interactions of GhSINA7, GhSINA8, and GhSINA9 were examined using an Y2H assay. The full-length cDNAs of *GhSINA7*, *GhSINA8*, and *GhSINA9* genes were fused independently to the DNA-binding domain (BD) bait vector pGBKT7 or the GAL4 activation domain (AD)-containing prey vector pGADT7, and their associations were determined. As shown in Figure 2B, each GhSINA interacted with itself and with the other two GhSINAs to form homo- and heterocomplexes, respectively.

### GhSINA Proteins Are Functional E3 Ubiquitin Ligases

**FIGURE 3:**
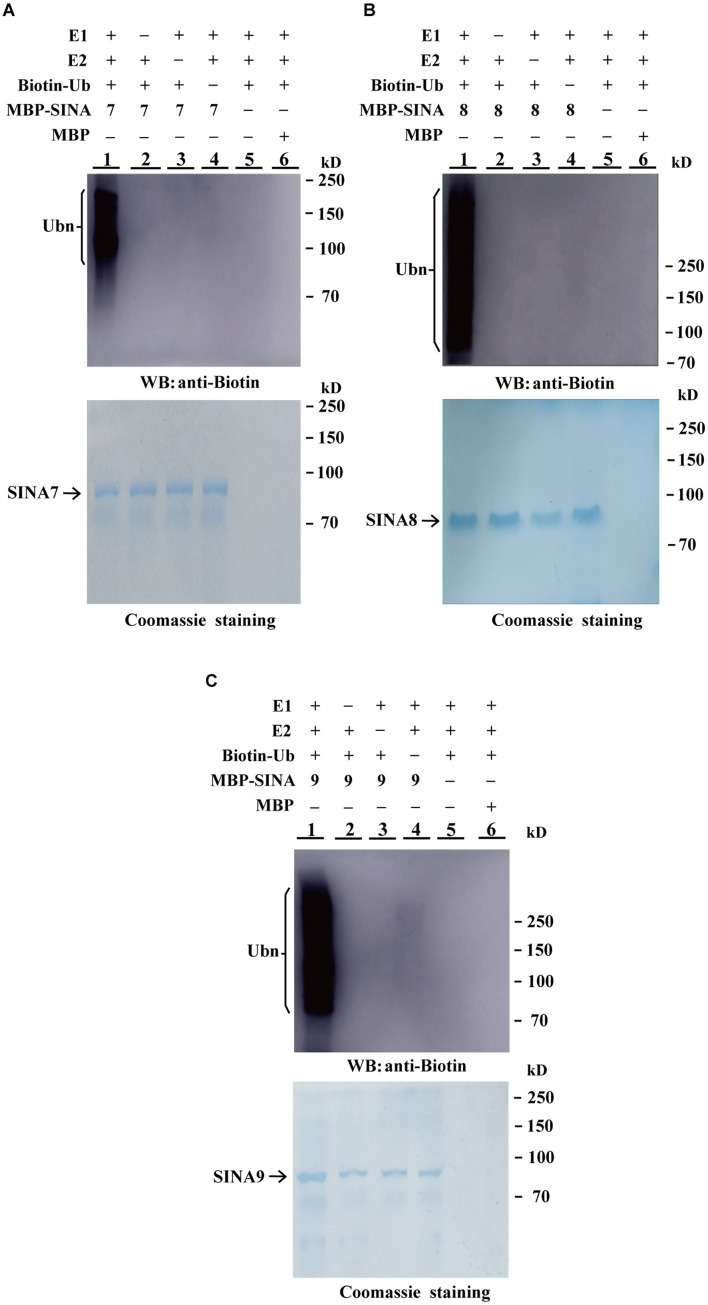
*In vitro* ubiquitination assays of GhSINA7, GhSINA8, and GhSINA9 proteins. **(A–C)** The E3 ubiquitin ligase activities of MBP-SINA7 **(A)** -SINA8 **(B)** and -SNA9 **(C)** fusion proteins were determined in the presence of E1, E2, and biotinylated ubiquitin (Bt-Ub) (Lane 1). Samples were resolved by 7.5% SDS-PAGE and determined by western blotting (WB) with an anti-biotin antibody. MBP was used as a negative control. Coomassie brilliant blue staining indicates equal amounts of SINA3 present in the reactions.

E3 ligases bind to E2 ubiquitin-conjugating enzymes and have the functional enzyme activity for self-ubiquitination. To determine whether the GhSINA7, GhSINA8, and GhSINA9 proteins, which are up-regulated in the presence of *V. dahliae*, have E3 ligase activities, we expressed SINAs fused to MBPs in *E. coli* and affinity-purified the MBP-SINAs from the soluble fractions. In the presence of human E1 (UBA1), E2 (UbcH5b), biotinylated-tagged ubiquitin (Bt-Ub) and MBP-SINAs, high molecular mass self-ubiquitinated smear ladders were detected using an anti-biotin antibody (Figures 3A–C, Lane 1), indicating that MBP-SINAs were ubiquitinated. There was no polyubiquitination signal when MBP-SINAs were replaced by MBP or when the E1, E2 or biotinylated-tagged ubiquitin was absent from the reaction (Figures 3A–C, Lanes 2–6). The results implied that the three SINA proteins possess E3 ubiquitin ligase activities.

### GhSINA Proteins Localized to the Cell Nucleus

**FIGURE 4:**
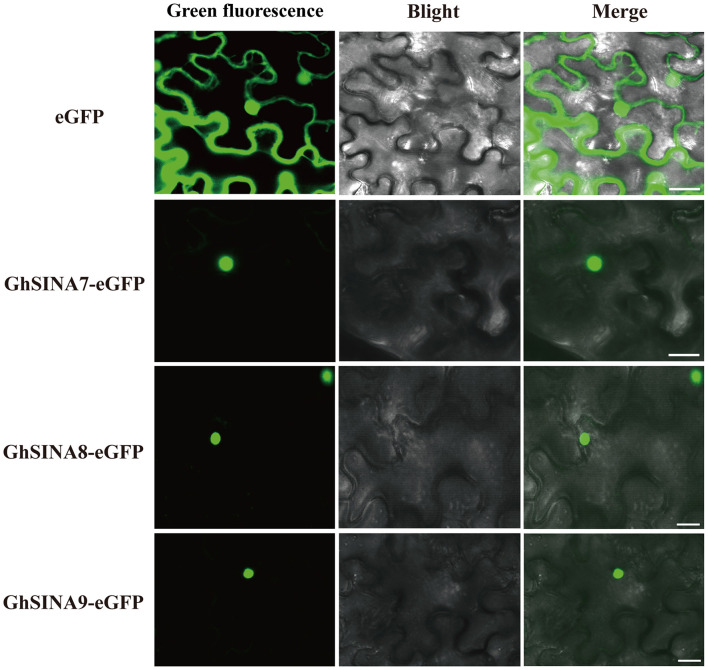
Subcellular localizations of the GhSINA7, GhSINA8, and GhSINA9 proteins in *N. benthamiana* epidermal cells as assessed by eGFP fusions. The fluorescence signals were visualized using confocal microscopy. Scale bars = 20 μm.

Ubiquitination usually occurs in the nucleus and cytoplasm to control nuclear and cytoplasmic proteins, respectively (Tanaka et al., 2005; Heck et al., 2010; Yoo et al., 2013). To determine the subcellular localizations of GhSINA7, GhSINA8, and GhSINA9 proteins, the *GhSINA7*/*8*/*9*-*eGFP* fusion constructs under control of the *CaMV 35S* constitutive promoter were transiently expressed separately in *N. benthamiana* leaves. As shown in Figure 4, the green fluorescence of free eGFP was observed in entire cells. Significantly, the three SINA proteins were present in nucleus, which is consistent with their functions in the ubiquitination pathway.

### *GhSINA* Overexpression Enhanced *V. dahliae* Tolerance in Transgenic Arabidopsis Plants

**FIGURE 5:**
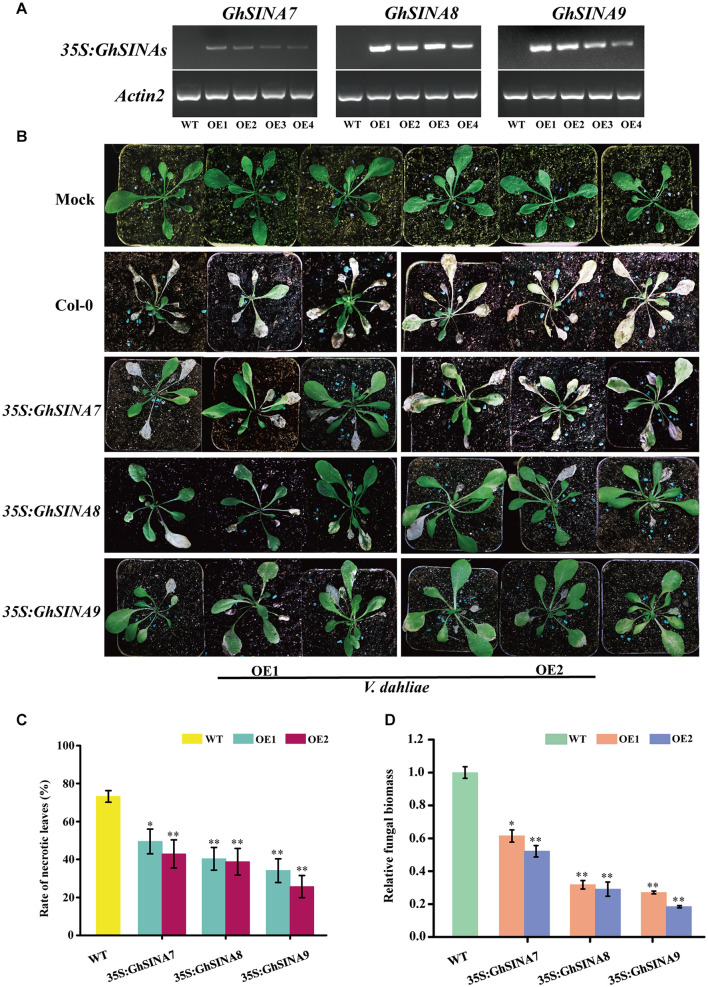
Enhanced disease tolerances of Arabidopsis plants independently overexpressing *GhSINA7*, *GhSINA8*, and *GhSINA9*. **(A)** Identification of transgenic Arabidopsis lines independently overexpressing *GhSINA7*, *GhSINA8*, and *GhSINA9* as assessed by RT-PCR. Arabidopsis *Actin2* was used as an internal control. **(B)** Symptoms of wild-type and *GhSINA7*, *GhSINA8*, and *GhSINA9* transgenic plants inoculated with *V. dahliae* for 10 days. **(C)** Disease rates of necrotic leaves in wild-type and transgenic Arabidopsis plants 10 days after *V. dahliae* inoculation. Error bars indicate the SDs of three biological replicates (*n* ≥ 32). Asterisks indicate statistically significant differences as determined by Student’s *t*-test (^∗^*P* < 0.05, ^∗∗^*P* < 0.01). **(D)** Quantitative measurement of fungal biomass. DNA of roots were extracted from plants 10 days post-inoculation by *V. dahliae*. A qRT-PCR analysis was employed to compare the DNA levels between the *V. dahliae* internal transcribed spacer (ITS) region and the *Actin2* gene of Arabidopsis. Error bars represent the SDs of three biological replicates. Asterisks indicate statistically significant differences as determined by Student’s *t*-test (^∗^*P* < 0.05, ^∗∗^*P* < 0.01).

An overexpression strategy was used to assess the functions of the *GhSINA* genes in defense responses. Owing to the long cotton transformation process, the model plant Arabidopsis was used in this experiment. More than 18 transgenic Arabidopsis lines heterologously overexpressing *GhSINA7*, *GhSINA8*, and *GhSINA9*, independently, were obtained. The two independent homozygous T_3_ lines with the highest *SINA* expression levels were selected for the phenotypic analysis (Figure 5A). The disease resistance levels of *SINA*-overexpression transgenic plants against *V. dahliae* were assessed at 18-day after planting. Disease symptoms were observed at 10 days post-inoculation, and overall, the leaves of transgenic plants showed more resistance to *V. dahliae*, with less wilting, chlorosis, early senescence and necrosis, than those of the wild-type (WT) Col-0 (Figure 5B). The disease tolerance of the *GhSINA7* transgenic line increased compared with WT, but it was much lower than in the *GhSINA8* and *GhSINA9* transgenic lines. The necrosis rates of diseased *GhSINA* transgenic lines were significantly lower than that of WT (Figure 5C). Moreover, the fungal biomass analysis confirmed that less fungal DNA accumulated in the roots of the transgenic plants, especially in the *GhSINA8* and *GhSINA9* transgenic lines (Figure 5D). Thus, the ectopic overexpression of *GhSINA*s conferred greatly enhanced *Verticillium* wilt resistance in Arabidopsis compared with WT.

### Silencing *GhSINA*s Impaired Cotton Resistance to *Verticillium* Wilt

**FIGURE 6:**
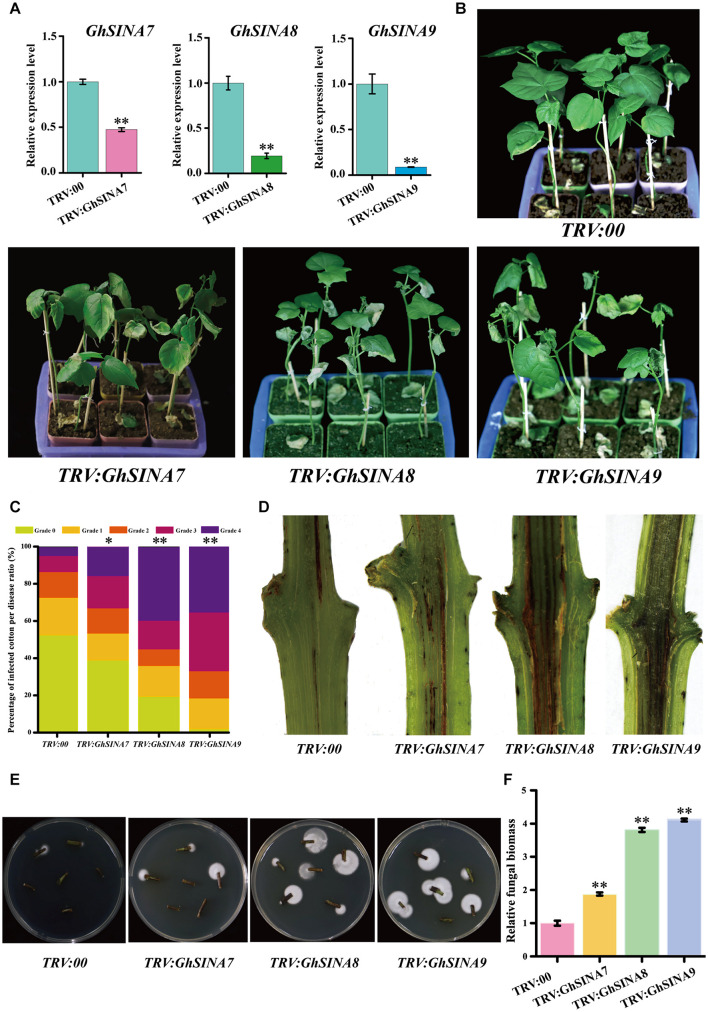
Silencing the *GhSINA7*, *GhSINA8*, or *GhSINA9* gene increased susceptibility to *V. dahliae* in cotton. **(A)** Transcript levels, as assessed by qRT-PCR, confirmed the silencing of *GhSINA7*, *GhSINA8*, and *GhSINA9* in different VIGS plants. Error bars represent the SDs of three biological replicates. Cotton *Histone3* was used as an internal control. Asterisks indicate statistically significant differences, as determined by Student’s *t*-test (^∗∗^*P* < 0.01). **(B)** Disease symptoms of *GhSINA7*-, *GhSINA8-*, and *GhSINA9*-silenced plants infected with *V. dahliae* strain V991 and photographed 14 days after inoculation. **(C)** The distributions of the disease rank levels. Grade 0: healthy leaves; Grade 1: leaves 0–25% wilted; Grade 2: leaves 25–50% wilted or chlorotic; Grade 3: leaves 50–75% chlorotic or necrotic; Grade 4: leaves dead or detached. The experiments were repeated three times using at least 40 seedlings per treatment. Asterisks indicate statistically significant differences as determined by the Wilcoxon rank-sum test (^∗^*P* < 0.05, ^∗∗^*P* < 0.01). **(D)** Darkened vascular discoloration of the dissected stems in *GhSINA7*-, *GhSINA8-*, and *GhSINA9*-silenced cotton plants compared with the controls (*TRV:00*) at 14 days after *V. dahliae* inoculation. **(E)** Fungal recovery assay. The surface-sterilized stem sections from control and silenced plants at 16 days after *V. dahliae* infection were cut and plated on potato dextrose agar medium. Photos were taken at 3 days after plating. **(F)** Relative fungal biomasses of silenced plants and controls 14 days after *V. dahliae* inoculation. The relative biomass represents a comparison of the DNA levels between the *V. dahliae* internal transcribed spacer (ITS) region and the cotton *Histone3* by qRT-PCR analysis. Values represent the means ± SDs from three biological replicates (^∗∗^*P* < 0.01, Student’s *t*-test).

The VIGS strategy, which is an efficient method for transient silencing of genes and widely used in cotton research (Li et al., 2019; Long et al., 2019), was employed to investigate the roles of *GhSINA* genes. Thus, we used VIGS to specifically silence each of the three *GhSINA* genes (homoeologous gene pairs silenced simultaneously) to study their functions during cotton responses to *V. dahliae*. The construct *TRV:GhCLA1*, which produces an obvious photobleaching phenotype when silenced, and empty *TRV:00* were used as the positive and mock controls, respectively. At 7 days after agroinfiltration, the leaves of cotton plants injected with *TRV:GhCLA1* displayed the expected photobleaching phenotype (Supplementary Figure 2). Additionally, the tender leaves of cotton seedlings infiltrated with different constructs were sampled for RNA isolation and qRT-PCR analyses. The expression levels of *GhSINA7*, *GhSINA8*, and *GhSINA9* dramatically decreased in their respective VIGS-treated plants compared with the *TRV:00* plants (Figure 6A). To investigate the specificity of the VIGS-mediated suppression of the three *SINA*s, the transcription levels of the non-targeted remaining two *SINA* genes, which shared high similarity levels (Supplementary Figure 1) with the silenced *GhSINA*-coding sequence, were detected in VIGS plants. The expression levels of two non-targeted SINA genes were not affected in each of the specifically silenced plants (Supplementary Figure 3).

Subsequently, the control and silenced plants were challenged by *V. dahliae*. Approximately 2 weeks later, the gene-silenced cotton seedlings, especially those containing *TRV:GhSINA8* and *TRV:GhSINA9*, displayed more severe leaf withering, yellowing and defoliation symptoms, and even death, than control plants (*TRV:00*) (Figure 6B). The DIs were calculated, and the results indicated that most of the *TRV:GhSINA8* and *TRV:GhSINA9* plants developed severe disease lesions (Figure 6C). Furthermore, more necrotic vascular tissues were found in *GhSINA8* and *GhSINA9*-silenced cotton plants than in *TRV:00* plants (Figure 6D). The fungal recovery assays confirmed that *GhSINA8-* and *GhSINA9*-silenced plants were subjected to more fungal colonization than control plants (Figure 6E). Correspondingly, the fungal biomasses were dramatically greater in *GhSINA8-* and *GhSINA9*-silenced plants than in control plants (Figure 6F). Collectively, these observations demonstrated that the silencing of *GhSINA7*, *GhSINA8*, or *GhSINA9* inhibited the plant immune system and enhanced the susceptibility to *V. dahliae*.

## Discussion

### Characterization of SEVEN IN ABSENTIA E3 Ligases in Cotton

The SINA E3 ubiquitin ligases are ubiquitous moderators that regulate plant growth and stress responses at the post-translational level (Shu and Yang, 2017; Zhang et al., 2019). Here, we report, for the first time, 24 SINA members containing highly conserved RING finger and SINA domains in upland cotton. The qRT-PCR analysis of cotton samples taken at 24 h after *V. dahliae* inoculation indicated that the *GhSINA7*, *GhSINA8*, and *GhSINA9* transcript levels were dramatically upregulated compared with uninfected controls (Figure 2A). A phylogram separated the *G. hirsutum* SINA7, GhSINA8, and GhSINA9 proteins into Group 1, which contains the ortholog of the *SINAT5* gene in Arabidopsis (Figure 1B). SINAT5 is a versatile regulator of plant developmental processes, including lateral root production, flowering time control and nodulation formation (Xie et al., 2002; Park et al., 2007; Den Herder et al., 2008). Thus, the SINA E3 ligases of Group 1 may, by modifying distinct substrates, have diverse functions in Arabidopsis and cotton.

The *SINA* genes encode C3HC4-type RING E3 ligases that are often active as dimers to sustain their own stability *in vivo* and perform different biological functions (Ning et al., 2011; Yang et al., 2015). The human homolog of SEVEN IN ABSENTIA (hSiah1) was found to oligomerize with itself and heterodimerize with other Sina and Siah proteins in upper eukaryotic cells (Depaux et al., 2006). Oligomerization of a protein does not seem to compete for binding of the substrates, but rather be involved in the formation of a higher ubiquitylation complex, assembling E2 ligases and proteins to be degraded (Depaux et al., 2006; Liew et al., 2010; Yang et al., 2015). Therefore, dimerization of SINA protein may allow simultaneous interaction with multiple proteins. In fact, the RING finger domain is required for the dimer formation of C3HC4 RING finger E3 ubiquitin ligases (Lee et al., 2009; Zhang et al., 2019). C3HC4 RING E3 ligases, such as Siah (Polekhina et al., 2002), RNF4 (Plechanovová et al., 2011), HAF1 (Yang et al., 2015), cIAP (Mace et al., 2008), SlSINA (Wang et al., 2018), MtSINA (Den Herder et al., 2008), and MdSINA (Li et al., 2020), can self-interact to form homodimers or heterodimers with other RING E3 ligases. Because two RING finger domains may be required to spatially accommodate E2 (Duncan et al., 2010; Pao et al., 2018), dimer formation probably depends on the ubiquitination activities of RING E3 ligases *in vivo*. Using the Y2H assay, we found that GhSINA7, GhSINA8, and GhSINA9 physically interact with themselves or with the remaining two GhSINAs to form homo- or heterodimers (Figure 2B). Thus, GhSINA E3 ubiquitin ligases may form homo- or heterodimeric complexes to perform the ubiquitination function.

### GhSINAs Are Positive Regulator Against *V. dahliae* Infection in Upland Cotton

Ubiquitin-mediated proteolysis plays a pivotal role in plant adaptability to changing environmental conditions, including exposure to a wide range of pathogens, such as bacteria, viruses and fungi, and insects. The RING-type E3 ubiquitin ligases control nuclear proteins targeted by ubiquitin-proteasome system activities during plant-pathogen interactions. In Arabidopsis, the E3 ligase MIEL1 negatively regulates hypersensitive cell death by ubiquitinating MYB30 (Marino et al., 2013). The transcription factor MYC2, as a master regulator targeted by E3 ligase PUB10, coordinates plant defense and development by repressing defense-related jasmonic acid- and ethylene-responsive genes, respectively (Boter et al., 2004; Lorenzo et al., 2004; Jung et al., 2015). BOI1 (MYB transcription factor BOS1-interacting E3 ligase 1) is involved in the regulation of signaling responses downstream of pathogen perception or cell death (Luo et al., 2010). The *Magnaporthe oryzae* effector AvrPiz-t represses the detection of pathogen-associated molecular pattern-triggered immunity by suppressing E3 ubiquitin ligase APIP6 in rice (Park et al., 2012). The receptor kinase XA21, regulated by E3 ligase XB3, provides resistance to rice bacterial blight disease caused by *Xanthomonas oryzae* pv. *oryzae* (Huang et al., 2013). In wild grapevine (*Vitis pseudoreticulata*), the RING-type E3 ubiquitin ligase EIRP1 mediates the degradation of transcription factor VpWRKY11 and attenuates the expression of jasmonic acid-responsive genes, which enhances resistance to fungal infections (Yu et al., 2013).

*Verticillium* wilt is caused by a highly aggressive fungal pathogen, resulting in severely reduced cotton fiber quality and yield worldwide (Fradin and Thomma, 2006; Cai et al., 2009). At almost 2 weeks after inoculation, susceptible cotton plants show visual disease symptoms of cotyledon wilting, leaf chlorosis and seriously stunted growth (Cox et al., 2019). Transgenes, VIGS and infection phenotypes are available approaches used to illuminate the molecular bases of cotton defense against *V. dahliae*. As shown in Figures 5, 6, the ectopic overexpression of *GhSINA7*, *GhSINA8*, and *GhSINA9* genes enhanced the tolerance of the transgenic Arabidopsis to *V. dahliae*. Knockdowns of *GhSINA7*, *GhSINA8*, and *GhSINA9* genes had compromised resistance to *V. dahliae*. These findings on the loss- and gain-of-functions of expressed *SINA*s in response to *V. dahliae* infection were consistent with *GhSINA*s being associated with plant defense against *V. dahliae*. Hereafter, stable *GhSINA-*overexpression and genome-edited transgenic upland cotton lines will be produced, which will help to ascertain the functions of GhSINAs in response to *V. dahliae*. Additionally, the specific substrates promoted by GhSINAs for degradation by mono- or polyubiquitination to mediate cotton responses to *V. dahliae* at the posttranscriptional level is still an urgent topic to investigate.

## Conclusion

In this study, we identified 24 *GhSINA* genes, and the expression levels of *GhSINA7*, *GhSINA8*, and *GhSINA9* were upregulated at 24 h after inoculation with *V. dahliae*. The three GhSINA proteins possessed E3 ubiquitin ligase activities and interacted with each other. The ectopic overexpression of *GhSINA7*, *GhSINA8*, and *GhSINA9* genes independently in *A. thaliana* increased the tolerance to *V. dahliae*, whereas silencing *GhSINA7*, *GhSINA8*, and *GhSINA9* genes independently enhanced susceptibility to *V. dahliae* in cotton, which suggested that the three genes act as positive regulators in defense responses against *V. dahliae* in cotton plants. The study increased our knowledge about the roles of *GhSINA*s in cotton biotic stress responses at the post-translational level.

## Data Availability Statement

The original contributions presented in the study are included in the article/Supplementary Material, further inquiries can be directed to the corresponding author/s.

## Author Contributions

WLi, XM, and DY conceived and designed the research. ZR, WLiu, XW, and JZ performed the experiments. FZ, HF, and JL provided the materials. ZR, WLiu, and MC analyzed the data. ZR, WLiu, and WLi prepared the figures and wrote the manuscript. DY and XM revised the manuscript. All authors have read and approved the final manuscript.

## Conflict of Interest

The authors declare that the research was conducted in the absence of any commercial or financial relationships that could be construed as a potential conflict of interest.

## Publisher’s Note

All claims expressed in this article are solely those of the authors and do not necessarily represent those of their affiliated organizations, or those of the publisher, the editors and the reviewers. Any product that may be evaluated in this article, or claim that may be made by its manufacturer, is not guaranteed or endorsed by the publisher.

## Footnotes

1
http://www.cottonfgd.org/

2
https://pfam.xfam.org/

3
https://www.ebi.ac.uk/interpro/

4
http://weblogo.berkeley.edu/logo.cgi

## Supplementary Material

Supplementary Figure 1 Multiple sequence alignments of the coding sequences of *GhSINAs*.

Supplementary Figure 2 The photobleaching phenotype of *TRV:GhCLA1* plants.

Supplementary Figure 3 The silencing efficiencies of the two non-targeted *GhSINA*s in different VIGS cotton plants.

Supplementary Table 1 The primers used in this study.

Supplementary Table 2 Protein sequences used in this study.
