# Supplementary figures and images for: SEVEN IN ABSENTIA Ubiquitin Ligases Positively Regulate Defense Against Verticillium dahliae in Gossypium hirsutum
Source: Front Plant Sci. 2021 Oct 29;12:760520. doi: 10.3389/fpls.2021.760520 (PMC8586545; doi:10.3389/fpls.2021.760520)

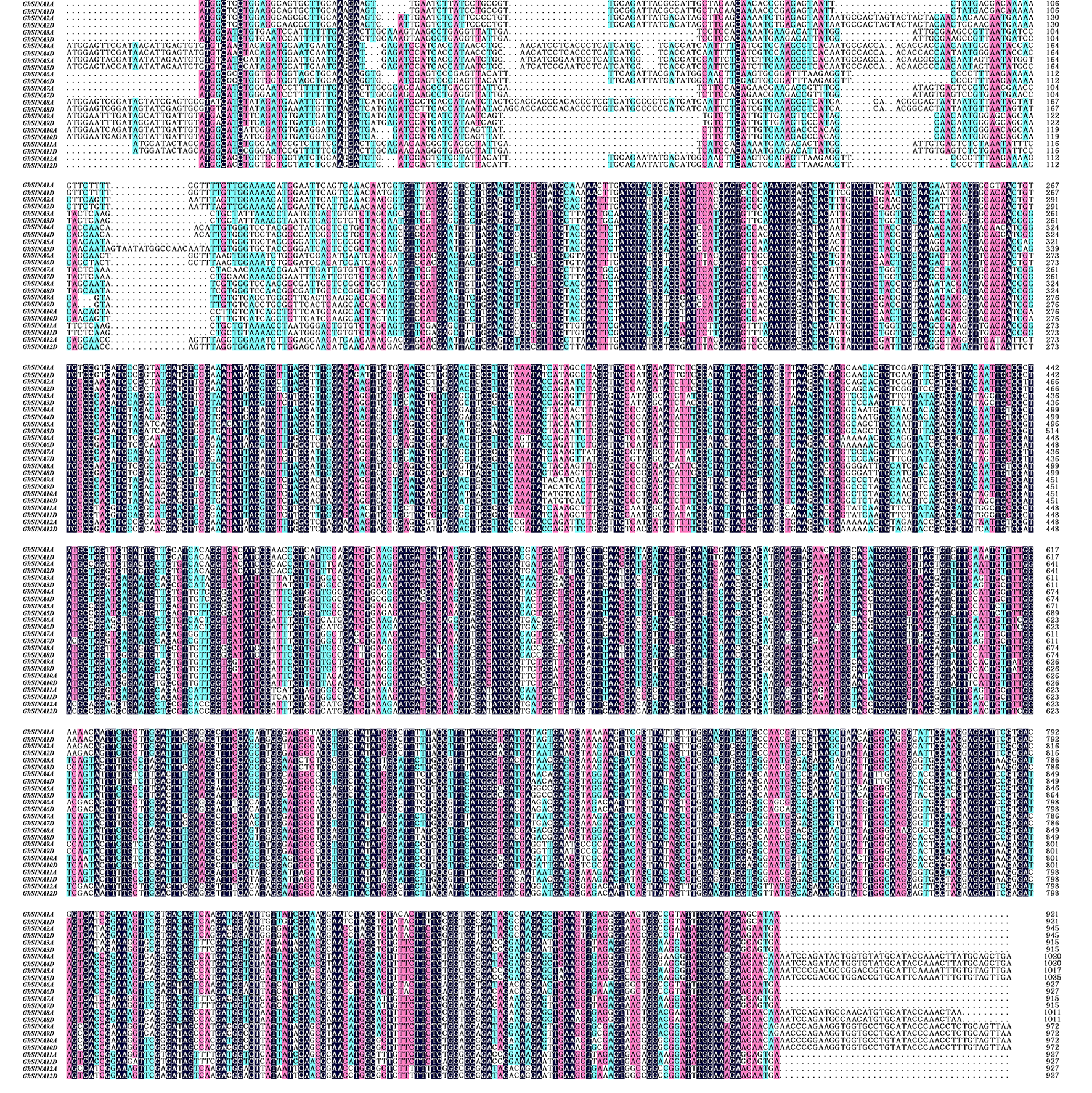

Supplement: Supplementary Figure 1 — Multiple sequence alignments of the coding sequences of GhSINAs. [file Image_1.TIF]

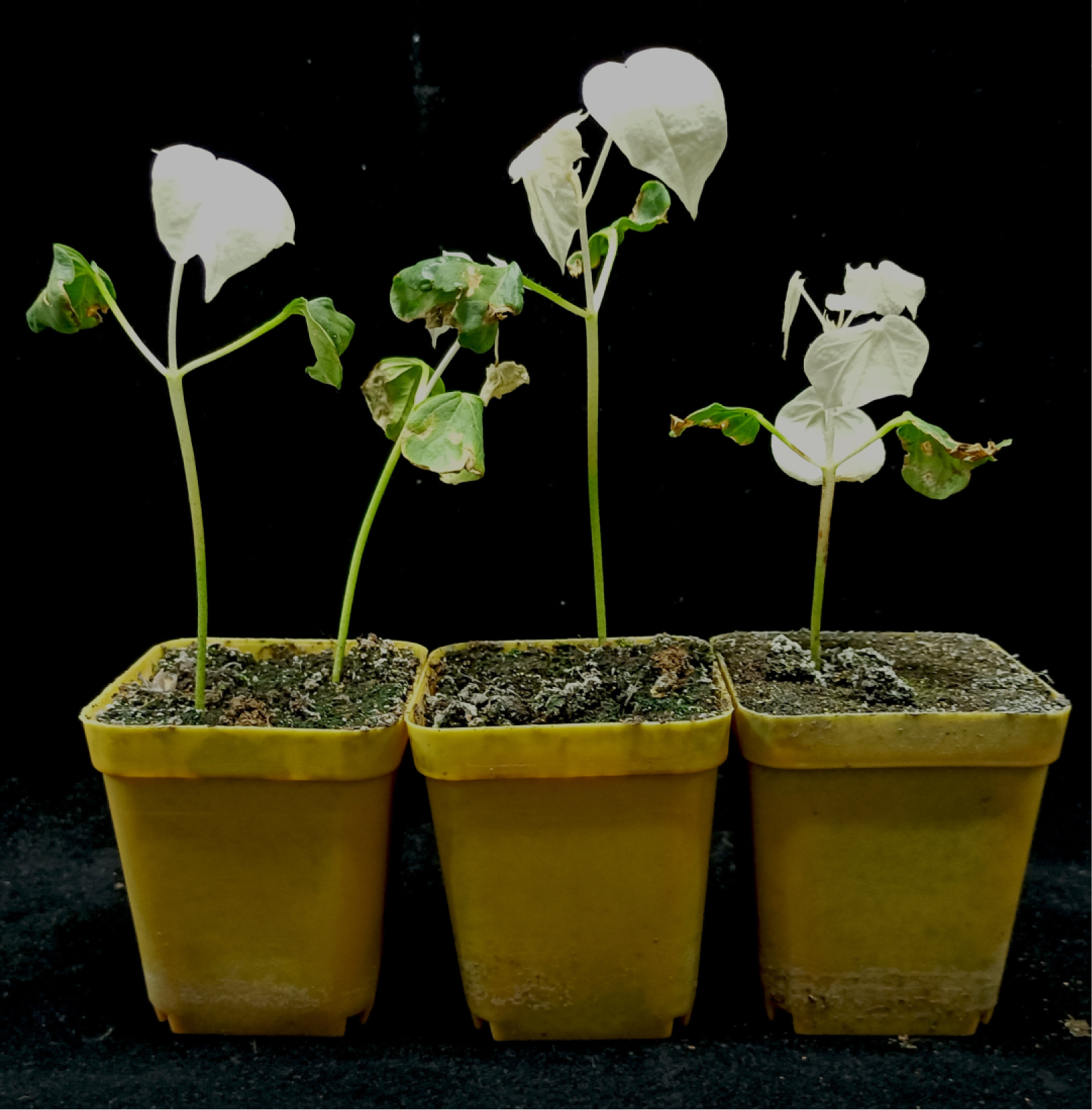

Supplement: Supplementary Figure 2 — The photobleaching phenotype of TRV:GhCLA1 plants. [file Image_2.TIF]

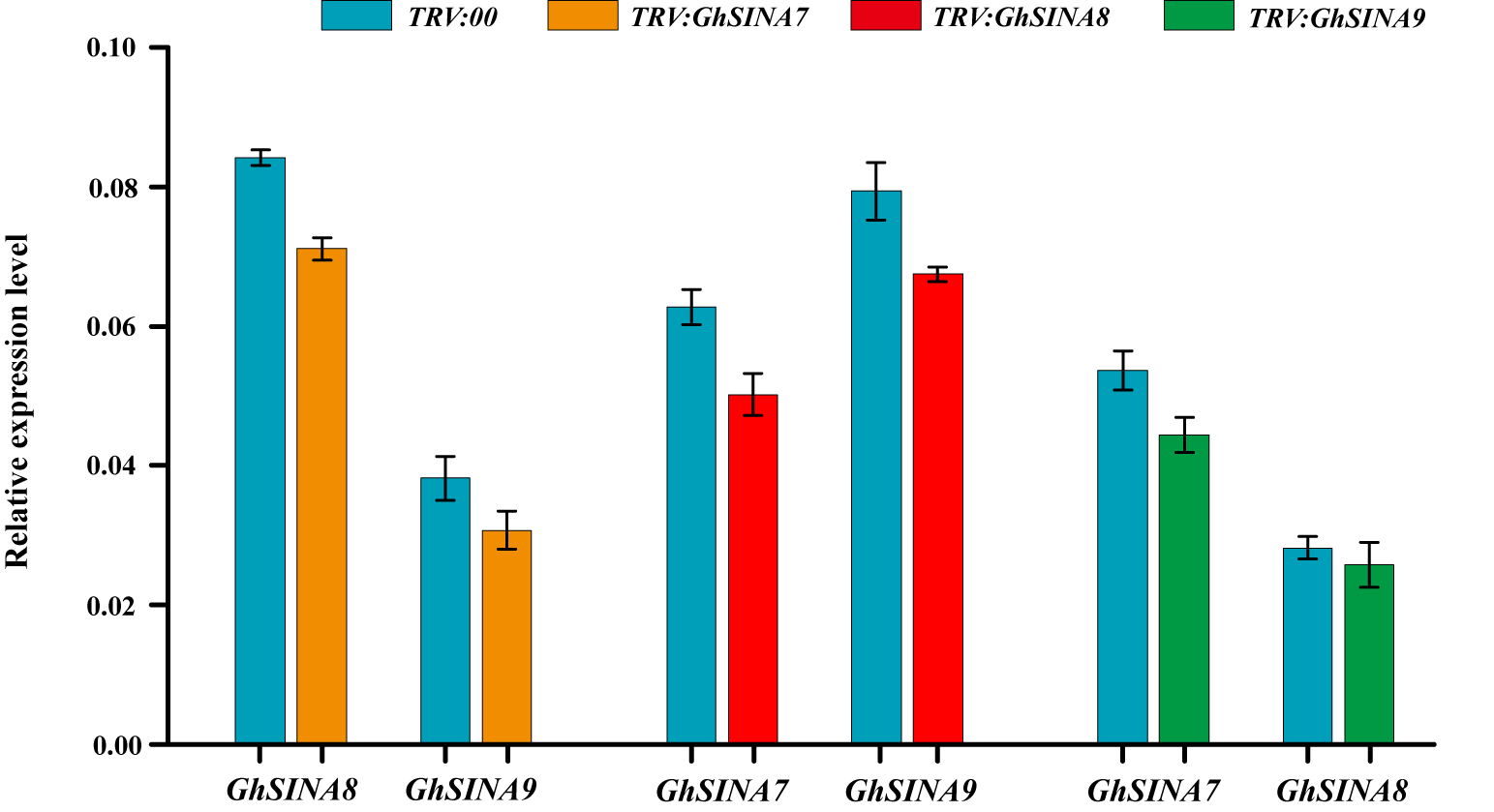

Supplement: Supplementary Figure 3 — The silencing efficiencies of the two non-targeted GhSINAs in different VIGS cotton plants. [file Image_3.TIF]
